# Within the Wards

**Published:** 1888-09-08

**Authors:** 


### Within the Wards.

### TUBERCULOSIS.

Among the latest researches into the cause and nature of tuberculosis are those of Dr. Sims Woodhead, Superintendent of the Laboratory of the Royal College of Physicians, Edinburgh. Dr. Woodhead states that it is now generally accepted that "all cases of rapid infective phthisis are the result of the action of the tubercle bacillus." He considers, however, that "certain lesions," presumably of a phthisical character, may be "produced by other and non-specific irritants." With both these propositions thoughtful and experienced physicians will be inclined to agree. Stonemasons' phthisis would seem to be a lesion, which, at the outset at any rate, may owe its origin to non-specific irritants rather than to bacillary action. But even here some of the lesions examined were tubercular, and bacilli have been demonstrated in certain new growths. Of course this may have been a mere coincidence in the particular cases examined; and the most that is thus proved is that in this variety of phthisis, as in other varieties, the bacillus is often associated with the disease, either as cause or effect, or as unexplained accompaniment. Dr. Woodhead referred to the researches of Dr. Julius Arnold "on the course taken by inhaled dust." Dust inhaled seems to produce, in many cases, a marked effect on the walls of the small bronchial tubes. Following such inhalation in certain instances there is observed a rapid proliferation of the alveolar epithelium, or, in untechnical language, a rapid multiplication of the cells which line the walls of the ultimate air-cavities of the lungs. This may terminate in a catarrh of those parts, that is in catarrhal pneumonia. Following this, pigment-granules may find their way into the lymph spaces around the ultimate air vesicles or cavities; thence, of course, into the lymphatic vessels, and finally into the glands connected with them at the root of the lungs. If such a course be possible for dust and pigment, it is argued that the tubercle bacillus may enter with the air, and acting as an irritant upon the alveolar epithelium, may pass through it and find its way by the lymphatics to the glands at the root of the lungs. Once there it is easy to imagine how rapidly and effectively bacilli pursue their work of destruction. Dr. Woodhead is decidedly of opinion that tuberculosis is a comparatively curable disease. In proof of this he adduces the cicatrices so often found in the lungs of old persons, and, in fact, of persons of every age. It is important to bear in mind that, according to the opinion of this and other eminent authorities, milk, air, and food may all convey the tubercle bacillus to the lungs. The best resistant of the bacillary poison is declared to be a vigorous condition of the general health. Bacilli attacking a healthy gland are probably harmless, but when the glands of weakly and delicate children are attacked the consequences are often rapid and fatal.

### SQUINTING AND WORMS.

That squinting may be caused by the presence of worms in the intestines has long been known; that if the worms are not removed the squinting may become a confirmed and almost incurable habit is not so generally recognised and acted upon as it should be. All children who squint should be placed under competent medical care, at least once, in order that a thorough examination may be made and a cure effected in every practicable case. Some time ago Mrs. D. consulted a well-known surgeon about her child's squint. There was at the time nothing to indicate that it was caused by worms. Indeed the mother was certain that it was not so caused—on what grounds it is not stated. The common symptoms of worms were at any rate absent. The sleep was not disturbed, the nose did not itch, there was no special craving for any particular kind of food, or for food at all. The patient was three years old, but yet could hardly speak intelligibly. When placed in a strong light the eyeballs turned upwards so completely that the "whites" alone were visible. The surgeon came to the conclusion, in opposition to the mother and all her friends, that the squint was caused by intestinal worms. Accordingly certain drugs were prescribed for the purpose of expelling the foreigners. The first dose brought away a large number of threadworms. A new drug was afterwards administered, which resulted in the expulsion of eighteen inches of tapeworm, and other portions came away on several successive days. Finally, in the course of the next fortnight, "five or six and twenty round worms," varying "from four to eight inches in length" were dislodged. The patient, who at the outset squinted so badly that she could hardly see, was completely cured of her squint in two months, and her sight was rapidly recovering. Here is a case in which skilled medical treatment was invaluable. The only comment called for is that such treatment should have been sought much sooner.

### SACCHARIN FOR DIABETICS.

Further experiments with saccharin as a sweetening agent for the use of diabetics seem to be required before its indiscriminate employment can be recommended. M. Worms, in introducing a discussion on the subject at the Académie de Medicine, reported by the *Therapeutic Gazette*, said that the most interesting application that he made of this remarkable product was that of enabling those who suffer from glycosuria to enjoy sweetened aliments without fear of increasing their malady. But he found saccharin was not very well borne by the patients to whom he gave it. Out of four treated only one was able to continue its use, the others suffering from gastric disturbance. Consequently he considered there would exist a certain amount of danger to the public if the drug were to be extensively used by confectioners, grocers, and others, in the sweetening of their products. Twenty grains of saccharin have a sweetening power equal to nine ounces of white sugar. M. Dujardin said he had not met with the accidents mentioned by his colleague. He gave as much as 120 grains to a dog without witnessing any ill effects, consequently saccharine was not toxic. However, it could not be considered an aliment, because it was found, after ingestion, in the urine unchanged. He agreed with M. Worms on the necessity of examining the subject more fully, and especially from a commercial point of view, as, if this product of tar were to be used by the trades mentioned, a very serious prejudice would be caused to one of their most important national industries.

### GALVANIC TREATMENT OF PARASITIC DISEASES OF THE SKIN.

Chicago has the credit of originating, or, at any rate, of encouraging this method of practice. Dr. Reynolds speaks favourably of its use in ringworm. Briefly, his method is as follows: The part to be operated upon must be thoroughly cleansed from scales, oily secretions, etc., and the skin completely bared. Both electrodes must be covered with moistened pads, the negative pole, thus prepared, being placed at a point conveniently near the place to be treated. The electrode of the positive pole is to be applied to the affected part; but first it must be well moistened with mercuric chloride solution, five grains to the ounce. It must be allowed to remain in contact a considerable time. In five weeks a cure is said to be effected. The first question which occurs to the medical reader is naturally this: Would not the mercuric chloride be quite as effectual in five weeks if it were well brushed in frequently with a stiff toothbrush?

